# Pulmonary artery thrombosis as the first presentation of Behçet’s syndrome: a case report and review of the literature

**DOI:** 10.1186/s13256-021-02931-1

**Published:** 2021-06-22

**Authors:** Ziyad Alakkas, Waad Kazi, Mohamed Mattar, Eman Abdul Wahhab Salem, Naglaa Fawzy Seleem

**Affiliations:** 1 Internal Medicine Department, King Abdul-Aziz Specialist Hospital, Taif City, Makkah Saudi Arabia; 2 Radiology Department, King Abdul-Aziz Specialist Hospital, Taif City, Saudi Arabia; 3 Faculty of Medicine, Benha University, Banha, Egypt

**Keywords:** Behçet’s syndrome, Vascular Behçet’s syndrome, Pulmonary thromboembolism, Pulmonary artery thrombosis, Splenomegaly

## Abstract

**Background:**

Behçet’s syndrome is a type of systemic chronic vasculitis of unknown etiology, frequently characterized by recurrent oral and genital ulcers and uveitis. It is less commonly characterized by arthritis and skin, vascular, and gastrointestinal involvements. Behçet’s syndrome affects various sizes of vessels by perivascular infiltration and vasculitis. Unlike other classic types of vasculitis, Behçet’s syndrome patients can present with both arterial and venous involvement. Although vascular Behçet’s syndrome is found in only around 15% of Behçet’s syndrome patients, it is the major cause of morbidity and mortality among them. Furthermore, although deep venous thrombosis has high incidence in Behçet’s syndrome patients, pulmonary artery thrombosis is an uncommon complication. Combining the findings of this and previous case reports of pulmonary artery thrombosis in Behçet’s syndrome patients, we sought to determine the best treatment options for pulmonary artery thrombosis in Behçet’s syndrome patients.

**Case presentation:**

We present the case of a 22-year-old Arabian male who was admitted to an emergency department with acute chest pain, dyspnea, and hemoptysis for 2 weeks. He gave a long history of recurrent oral and genital ulcers for the last 4 months but without seeking medical advice. Spiral computed tomography showed arterial filling defects with a pulmonary nodule for which the presence of a pulmonary artery aneurysm ruled out. The lung perfusion scan showed multiple pulmonary perfusion defects. After excluding common infectious diseases such as tuberculosis and brucellosis, a diagnosis of Behçet’s syndrome with pulmonary artery thrombosis was made. Steroids with enoxaparin were initiated. The patient was discharged later on prednisolone (tapering dose) with adalimumab and apixaban. He was on regular follow-up for the next 9 months.

**Conclusions:**

Vascular involvement in Behçet’s syndrome is a major contributor to morbidity and mortality of Behçet’s syndrome patients. Consequently, early detection of vascular involvement has a major impact on the prognosis of patients with Behçet’s syndrome.

## Background

Behçet’s syndrome (BS) is a chronic systemic form of relapsing remitting vasculitis. Vasculitis involves multiple organs, including the skin, joints, eye, mucosa, veins, arteries, nervous and gastrointestinal systems, and others. BS is usually diagnosed between the third and fourth decade of life, being slightly more common in males compared with females, and presents with more serious features in young adult males [1, 2]. BS is more frequent in the Middle East, the Mediterranean, and the Far East regions. Prevalence of BS varies by geographical area; Turkey has had the highest number of reported cases at 420 cases per 100,000 persons, followed by northern China, Iran, and Korea. In contrast, the prevalence in Saudi Arabia is around 20 cases per 100,000 people [3]. The classic clinical presentation of BS is painful oral aphthous ulcers, genital ulcers, and ocular involvement, symptoms that together are called the triple-symptom complex. However, other symptoms may be initially involved as well. After ruling out other differential diagnoses, diagnosis of BS is based on diagnostic clinical criteria as determined by the International Study Group (ISG) [1, 4]. BS patients need physicians from different subspecialties to care for their acute and chronic health issues.

Vascular involvement in BS patients has major impacts on their quality of life. Vascular Behçet’s syndrome (VBS) is unique in that it affects both the venous and arterial systems. The frequency of vascular complications in Behçet’s syndrome is around 12–15% [5, 6], being more common in young males. Most VBS patients (~ 75%) experience their first vascular event within 5 years of disease onset, 10.8% of whom present a vascular event before fulfilling the ISG criteria. In a previous study, most patients (87%) who presented initial vascular involvement exhibited deep venous thrombosis (DVT), while only 1 out of 882 patients had pulmonary artery involvement [5]. Venous thrombosis in BS can severely impact patient health, increasing the risk of developing Budd–Chiari syndrome and resulting in a 10% mortality rate in BS patients. Arterial involvement leads to an even higher risk (three times) of mortality in BS patients by increasing the risk of developing severe clinical issues such as arterial aneurysms [2].

There are several forms of venous thrombosis in BS, including pulmonary artery thrombosis [5]. DVT is the most common venous thrombosis in BS patients. Although incidence of DVT in Behçet’s syndrome is high, pulmonary artery thrombosis (PAT) is considered a rare complication. There have been a couple of reported cases of patients with Behçet’s syndrome who exhibited pulmonary thrombosis during BS pathogenesis (Table 1). The most common presenting symptom of PAT is hemoptysis [6]. Conventional chest radiography is typically used to initially assess pulmonary symptoms and signs of BS. Spiral computed tomography (CT) is considered an excellent next step as it is a noninvasive procedure [5]. Pulmonary artery thrombosis may accompany aneurysms, which may lead to infarction, atelectasis, and hemorrhage.

**Table 1:** Studies of pulmonary thrombosis in patients with Behçet’s syndrome

No.	Study	Type	Year	Ref.
1	An adult male presenting with bloody sputum diagnosed as BS with pulmonary embolism	Case report	2020	[18]
2	A young male chest pain diagnosed as BS with pulmonary embolism. Discharged on steroids and anticoagulation	Case report	2019	[19]
3	Massive bilateral pulmonary thromboembolism on second day after total hip arthroplasty. Methotrexate withheld 15 days before surgery and restarted second day postoperatively	Case report	2019	[20]
4	Adult male with BS for 12 years presented with PTE manifestations. Discharged with monthly doses of CYC, steroids, and warfarin	Case report	2018	[21]
5	Young male developed PE and started on apixaban; he presented again with chest pain, so diagnosed with pulmonary vasculitis Behçet’s syndrome; steroids were added and apixaban continued	Case report	2018	[22]
6	3 out of 7 Behçet’s syndrome patients with pulmonary vascular disease were diagnosed as PE in Shanghai Pulmonary Hospital from 2009 to 2016.They received immunosuppressive agent and anticoagulant	Retrospective	2017	[23]
7	Adult male with DVT on warfarin presented again with PE. Later diagnosed as Behçet’s syndrome. Discharged on azathioprine and steroids	Case report	2016	[24]
8	Total of 766 patients with BS, of whom 93 developed thrombosis; 14 patients (15.1% of thrombosis cases) developed PTE	Retrospective	2014	[25]
9	13 (28%) out 47 Behçet’s syndrome patients with pulmonary involvement had isolated pulmonary artery thrombus	Cohort study	2012	[13]
10	Total of 41 BS patient died; 3 patients were PE	Retrospective	2010	[26]

BS: Behçet’s syndrome, PTE: Pulmonary thromboembolism, CYC: cyclophosphamide, PE: Pulmonary embolism, DVT: Deep venous thrombosis

The reported frequency of gastrointestinal involvement ranges from 1% to 30%. Symptoms include nausea, abdominal pain, and diarrhea, with complications such as fistulas and bowel perforation [7]. Splenomegaly is not a typical characteristic of BS; only a few case reports have found splenomegaly in BS patients [8].

We report here in the case of a young male not known to have any chronic diseases who presented with recurrent aphthous ulcers, chest pain, hemoptysis, and splenomegaly. We discuss the challenges of diagnosis and treatment of PAT in Behçet’s syndrome. Written consent was secured, and ethical approval by the Institutional Review Board (IRB) at Taif Health Affairs and the Medical Research Unit at King Abdul-Aziz Special Hospital was obtained.

## Case report

A 22-year-old Saudi Arabian male patient, not known to take medication or have any chronic medical diseases, was admitted to our hospital. For 12 days, he exhibited acute pleuritic chest pain, exertional dyspnea, and hemoptysis associated with subjective fever and sweating. No lower limb pain or swelling was observed. The patient had a history of recurrent painful oral ulcers (one to four small ulcers) over 3–5 days for 4 months, which spontaneously disappeared. The patient also exhibited frequent recurrent painful genital ulcers, mainly on the scrotum. Although the patient was not on a specific diet, he experienced significant weight loss of nearly 8 kg (13% of total body weight) over 6 months. No significant family history of rheumatological or autoimmune diseases, or similar conditions was noted. He was single, lived on a farm, and frequently ingested raw milk.

During examination, the patient was fully conscious and was neither disoriented nor in distress. The patient’s blood pressure was 126/70 mmHg, with heart rate of 110 beats per minute, respiratory rate of 20 per minute, oxygen saturation of 95% on ambient air, temperature of 37.4 °C, and body mass index of 19 kg/m^2^. The patient’s mouth exhibited three small, rounded tender ulcers on the oral mucosa and at the base of the tongue. The genitals exhibited one minor tender nonpurulent ulcer on the scrotum.

**Fig. 1:**
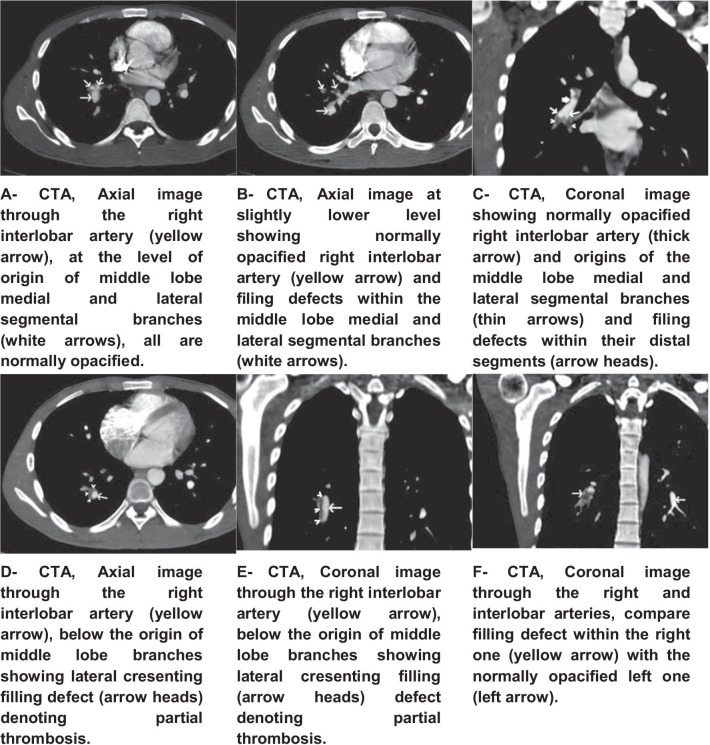
Spiral chest CT scan angiography

The patient’s laboratory data were as follows: white blood count of 13.7 × 10^9^/L (neutrophils 68%, lymphocytes 25%), hemoglobin level of 11.8 mg/dL, platelet count of 337 × 10^9^/L, coagulation profile normal (INR 1.1), d-dimer of 0.04 mg/L, erythrocyte sedimentation rate (ESR) of 78 mm/hour, and C-reactive protein (CRP) of 10.9 positive. Kidney function tests, liver function tests, and electrolytes were within the normal range. Screening tests for antinuclear antibody (ANA), anti-double stranded deoxyribonucleic acid (anti-dsDNA), anti-cardiolipin, hepatitis B virus (HBV), hepatitis C virus (HCV), *Brucella* stiter and culture, and human immunodeficiency virus (HIV) were negative. The tuberculin skin test was 8 mm. Screens using three samples of sputum for acid-fast bacilli, Tuberculosis (TB) polymerase chain reaction (PCR), and GeneXpert were negative. Electrocardiograph (ECG) detected sinus tachycardia. Chest X-ray and transthoracic echocardiography (TTE) results were unremarkable. Fundus examination found bilateral vitreous degeneration and acute posterior uveitis. Abdominal ultrasound with Doppler showed the homogeneous echo pattern of a normal liver with no dilatation of the intrahepatic biliary radicles. The patient exhibited a normal portal vein (PV) and common bile duct (CBD) diameter. The spleen showed a bulky enlarged shape with span of around 13 cm and a homogeneous echo pattern. There was normal patency and color of Doppler signal flow of the portal, superior mesenteric, and splenic veins, with no evidence of thrombosis. Vasculitis was detected via fundus fluorescein angiography (FFA). CT angiography showed filing defects on the right interlobar and lower lobar segmental arteries. Lateral crescent filling defects were seen, denoting partial thrombosis. A right apical segment solitary nodule with size of 2.7 mm was also detected. Pulmonary artery aneurysms were ruled out (Fig. 1).

**Fig. 2:**
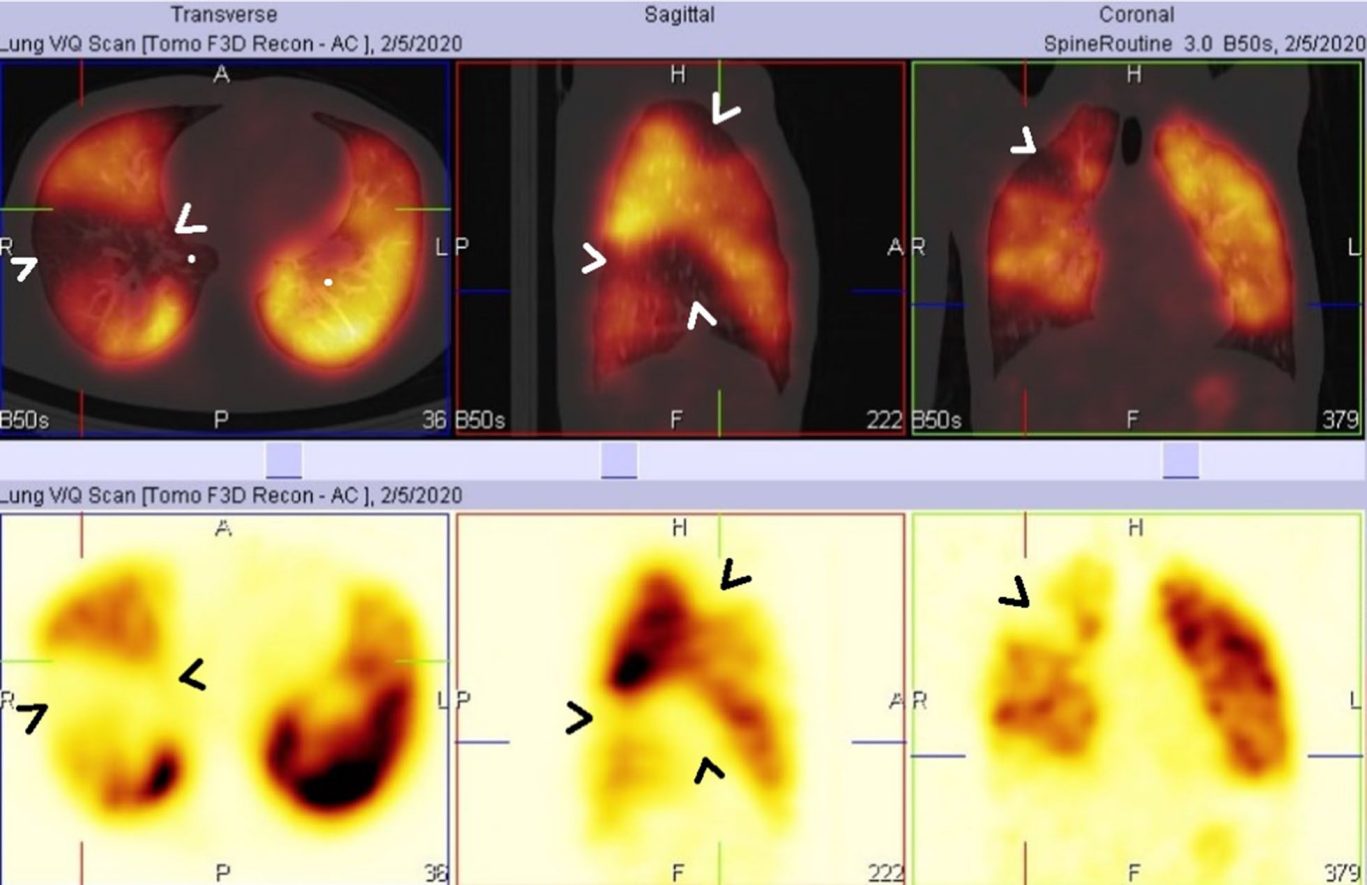
Axial and reconstructed views of SPECT-CT slices show multiple right lung middle lobe (medial and lateral segmental), upper lobe anterior segment, lower lobe anterobasal, laterobasal, and posterobasal segment perfusion defects (arrow heads)

Pulmonary perfusion scan (V/Q scan) showed multiple pulmonary thrombi in the right lung middle lobe, upper lobe anterior segment, and lower anterior basal lobe, while laterobasal and posterobasal segments exhibited perfusion defects (Fig. 2).

After ruling out common infectious diseases including tuberculosis and brucellosis, the patient was diagnosed with BS complicated by PAT based on clinical and radiological findings. Spiral CT ruled out pulmonary artery aneurysm, which is as a common vascular complication. The patient received pulse intravenous steroid therapy with methylprednisolone (1 g for 3 days) and a subcutaneous dose of enoxaparin (1 mg/kg twice per day). The patient’s clinical condition started to improve. The patient was discharged on prednisolone (tapering dose of 50 mg/day), colchicine (0.5 mg two times per day), adalimumab SubQ (40 mg every other week), and apixaban (5 mg twice per day). The patient was also to be reevaluated in rheumatology, pulmonology, and ophthalmology clinics on a monthly basis. The patient was in complete remission at 9-month follow-up. ESR and CRP were repeated after 3 months and showed normal ranges. Spiral CT was performed 9 months later. There was no evidence of aneurysms, intracardiac thrombus, or new pulmonary thrombi. Patient was screened for thrombophilia including anti-thrombin III, protein C, protein S, lupus anticoagulant, and anti-cardiolipin antibody-Immunoglobulin-G (Ig-G), and all were within normal. No complications were observed during follow-up.

## Discussion

Behçet’s syndrome is a type of systemic vasculitis that affects vessels of various sizes and multiple organ types. Vascular complications have a major impact on the morbidity and mortality of BS patients. The prevalence of vascular involvement in BS varies by country, where the highest rates have been reported in Morocco (62.3%) [9] but lower rates in Japan (7.4–8%) [10]. Venous involvement is more common (nearly 30% of patients) than arterial involvement (3–5%) [1]. Although isolated deep venous thrombosis is the most common form (67%) of major vascular disease in Behçet’s syndrome [5], PAT is considered a rare complication of Behçet’s syndrome. However, PAT is one of the main causes of morbidity and mortality in BS patients [11, 12]. Its etiology is mainly related to the disease behavior, including recurrent blood vessel inflammation that strongly adheres to vessels and makes embolization difficult, dissimilar to classic thrombi [6]. The thrombi are usually tightly adhered to the vessel walls and may be confused with embolisms, so it is important to initially image the vascular system with Doppler US and utilize advanced radiological methods such as CT venography (CTV), magnetic resonance angiography (MRA), and magnetic resonance venography (MRV) for appropriate diagnosis. CT angiography yields a lot of information regarding anatomy but has limitations, especially in the early stages of disease pathogenesis [1].

In this case, a young adult Arabian male who previously had recurrent oral and genital ulcers and did not seek any medical advice was finally admitted to the ED after exhibiting a vascular complication of Behçet’s syndrome. Since the patient had a history of fever, weight loss, and hemoptysis, tuberculosis was highly suspected, and the patient was admitted to an isolation room. However, tuberculosis was ruled out. He also had the classic BS manifestations of oral and genital ulcers plus ocular symptoms. Since the patient also presented with acute chest pain, dyspnea, and hemoptysis, vascular involvement of either pulmonary artery aneurysm or pulmonary artery thrombosis was suspected. Spiral CT showed pulmonary artery thrombosis with a pulmonary nodule and a pulmonary artery aneurysm ruled out. Later, V/Q scan confirmed presence of thrombosis. A diagnosis of pulmonary artery thrombosis was made before fulfilling the criteria of BS. Based on pathology behavior, autopsy results, and radiological findings of previous studies, pulmonary artery thrombosis has been a more consistent component of BS than pulmonary thromboembolism [5]. Pulmonary nodules are commonly associated with pulmonary artery involvement in BS patients [13].

The primary treatment objectives for BS patients exhibiting vascular involvement are to suppress inflammation and prevent further complications as soon as possible. There are no standard guidelines for the management of venous thrombosis in Behçet’s syndrome. High doses of steroids, azathioprine, and cyclophosphamide are the main immunosuppressants used to treat pulmonary thrombosis. Treating patients via pulse IV steroid therapy with methylprednisolone followed by oral prednisolone has been common practice. Administration of anti-tumor necrosis factor (TNF) monoclonal antibodies with anticoagulation could be considered for treating patients with refractory venous thrombosis who are at low risk of bleeding. However, pulmonary artery aneurysms should be ruled out before initiation of anticoagulation [14]. Anti-TNF could also be used instead of cyclophosphamide [1]

The cornerstone treatment for PAT in Behçet’s syndrome is immunosuppression. However, using anticoagulants in pulmonary thrombosis is controversial, as it may worsen the symptoms [11], but it is considered necessary in other situations [15]. A previous metaanalysis, which compared three groups utilizing different types of treatment regimens, determined that immunosuppression with anticoagulation in Behçet’s syndrome had better outcomes in terms of recurrence of DVT compared with anticoagulation treatment alone. In the same study, there were no significant benefits to treating patients with immunosuppression and anticoagulation as compared with immunosuppression treatment alone [16, 17]. Based on two studies, bleeding complications have been reported in 2.4–4.5% of anticoagulated patients [17]. To date, no comprehensive studies have been done to determine the appropriate treatment method for BS patients exhibiting PAT or venous thrombosis, so more studies are needed to cover this information gap.

Splenomegaly is an atypical manifestation of BS [7, 8]; the possible causes of splenomegaly, such as infectious agents (e.g., brucellosis or TB) or thrombosis-like Budd–Chiari syndrome, were ruled out in our case.

## Conclusions

Vascular involvement in Behçet’s syndrome is a major contributor to morbidity and mortality of such patients. Early detection of vascular involvement has a major impact on the prognosis of patients with Behçet’s syndrome. The mainstay treatment of pulmonary artery thrombosis is immunosuppression and anticoagulation, but the effectiveness of anticoagulation is debatable.

## Data Availability

Not applicable.

## Abbreviations

BS: Behçet’s syndrome
VBS: Vascular Behçet’s syndrome
PTE: Pulmonary thromboembolism
DVT: Deep venous thrombosis
TB: Tuberculosis
CTA: Computed tomography angiography
SPECT CT: Single-photon emission computerized tomography
CT: Computed tomography
